# Impact of model resolution on the representation of the wind field along Nares Strait

**DOI:** 10.1038/s41598-021-92813-9

**Published:** 2021-06-24

**Authors:** G. W. K. Moore

**Affiliations:** 1 Department of Physics, University of Toronto, Toronto, ON Canada; 2 Department of Chemical and Physical Sciences, University of Toronto Mississauga, Mississauga, ON Canada

**Keywords:** Atmospheric dynamics, Cryospheric science

## Abstract

Nares Strait is a major pathway along which multi-year sea ice leaves the Arctic, an ice class that has seen a recent dramatic reduction in extent. The winds that blow along the strait play an important role in modulating this ice export as well as in establishing the Arctic’s largest and most productive polynya, the North Water, that forms at its southern terminus. However, its remote location has limited our knowledge of the winds along the strait. Here we use automatic weather station data from Hans Island, in the middle of the strait, to assess the ability of a set of atmospheric renalyses and analyses with a common lineage but with varying horizontal resolution to represent the variability in the wind field. We find that the flow is highly bidirectional, consistent with topographic channeling, with the highest wind speeds from the north and that a model resolution of ~ 9 km is required to capture the observed variability. The wind field at Hans Island is also found to be representative of variability in the flow along much of Nares Strait.

## Introduction

**Figure 1:**
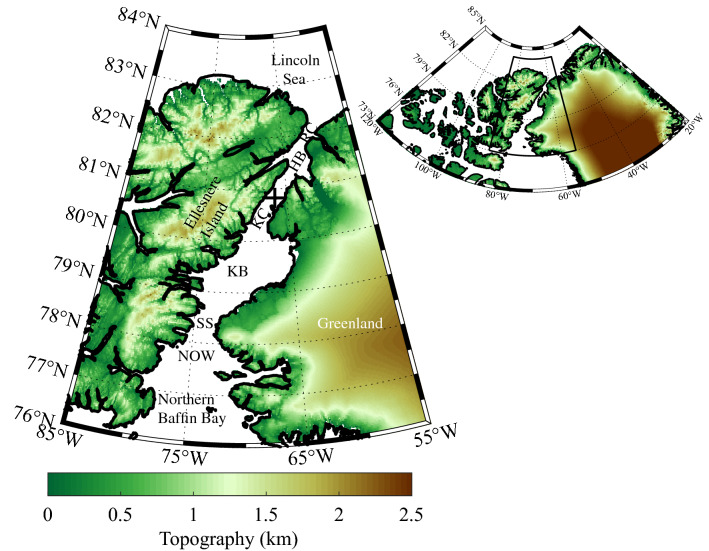
Topography (km) of the Nares Strait region as represented in the GEBCO dataset with the inset showing the location of the region with a larger context. The location of Hans Island is indicated by the ‘ + ’. The locations of the North Water Polynya (NOW), Smith Sound (SS), the Kane Basin (KB), the Kennedy Channel (KC), the Hall Basin (HB) and the Robeson Channel (RC) are also indicated. Figure created using Matlab R2019b (https://www.mathworks.com).

Nares Strait is the ~ 600 km long and ~ 40-80 km wide strait that connects the Arctic Ocean’s Lincoln Sea to northern Baffin Bay (Fig. 1). The steep topography along both sides of the strait (Fig. 1) as well as the generally higher sea-level pressures over the Lincoln Sea as compared to northern Baffin Bay results in a tendency for northerly flow down the strait^1^. The wind field in such a narrow channel is typically ageostrophic and controlled by the pressure gradient in the along-strait direction^2^. This channeling is enhanced by the common occurrence of low-level temperature inversions in the region^3^ that serve to inhibit vertical motion^4^.

The oldest and thickest sea ice in the Arctic is situated to the north of Nares Strait^5^ and the strait is a major pathway for the export of this important ice class out of the Arctic^6^. The wind field along Nares Strait with its preference for northerly flow plays an important role in the export^1^. Ice arches that form most winters along Nares Strait can result in the cessation of this ice transport for months at a time^6,7^.

The presence of these arches also contributes to the largest and most productive polynya in the Arctic, the North Water (NOW) that forms at the southern end of Nares Strait (Fig. 1), in the vicinity of Smith Sound^8^. With an arch present, ice motion along the strait is inhibited and the northerly flow down Nares Strait, that accelerates in the exit region of Smith Sound, is able to remove sea ice thus contributing to the maintenance of the polynya’s open water^9^.

There is evidence that the ice arches along Nares Strait are becoming less stable^7^ and that the area of the NOW is increasing^10^. There is also evidence that anomalous winds play a role in the collapse of these arches^11^. In addition, the Arctic is undergoing a transition towards a younger^12^ and thinner^13^ ice pack that is leading to a loss of multi-year ice from the Arctic^14^. There has also recently been an increase in both ice area and ice volume fluxes along Nares Strait^7^ that has been suggested to contribute to the loss of multi-year ice from the Arctic^6^.

The remote and data sparse nature of the region implies that there is still considerable uncertainty regarding the characteristics of the wind field that limits our ability to fully understand its role in ice export and its contribution to the formation of the NOW as well as the wind’s role in the changes that are occurring in the region.

Limited wind observations from the Smith Sound region confirm the preference for northerly flow with mean wind speeds on the order of 6–8 ms^−1^^8,15^. However observations extending back to the 1860s indicate that long-lived gales, events characterized by northerly surface wind speeds in excess of 20 ms^−1^, are a common occurrence in the Smith Sound region during the winter months^16^.

Wind observations farther north along Nares Strait are even rarer. In April 2005, an ice camp established along the Kennedy Channel was destroyed by northerly winds in excess of 25 ms^−1^^17,18^. During the winter of 1871–1872, the United States North Polar Expedition’s ship was trapped in ice at an exposed site in Hall Basin and the science party regularly observed wind speeds in excess of 20 ms^−1^ with a preference for northerly flow^19^. In the vicinity of Robeson Channel, meteorological observations were made at Fort Conger from August 1881 to August 1883 during the First International Polar Year by members of the United States Expedition to Lady Franklin Bay^20^. These observations indicated the preference for bidirectional wind flow, either in the NNE or SSW direction, along Robeson Channel. There were numerous events, with both directionalities, where the wind speeds were in excess of 15 ms^−1^^20^.

**Figure 2:**
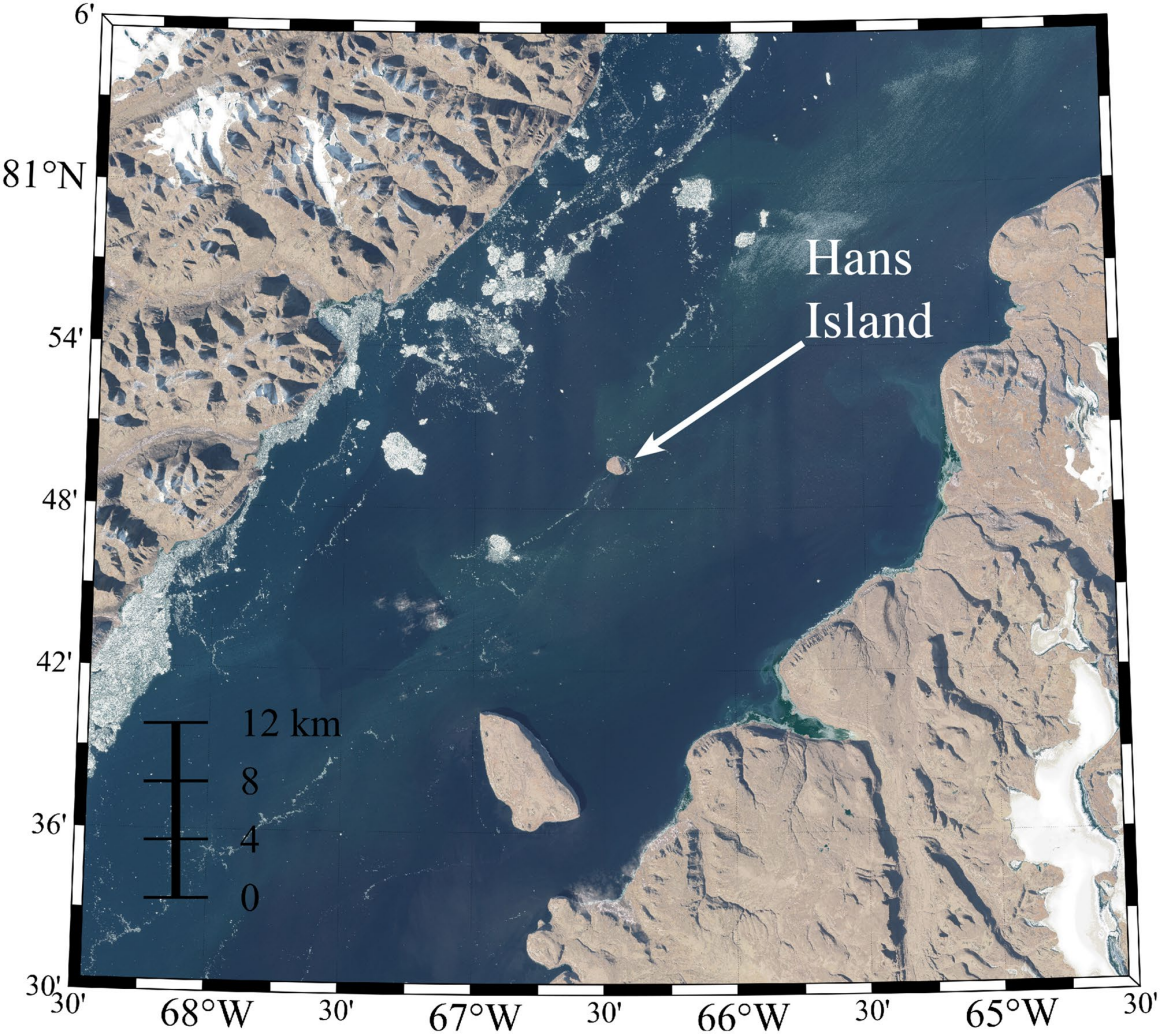
True-color high spatial resolution (~ 10 m) Sentinel-2 imagery of the Kennedy Channel in the vicinity of Hans Island on August 7 19:39 GMT 2020. Figure created using Matlab R2019b (https://www.mathworks.com).

Hans Island (80^o^49′35'N, 66°27′35'W), jointly claimed by both Canada and Denmark, is situated in the middle of the Kennedy Channel, one of the narrower sections of Nares Strait (Figs. 1 and 2). Since 2008, an automatic weather station (AWS) has been operating on this island^21^ with data currently available from 2016 onwards. This paper represents the first use of this data to characterize the wind field along Nares Strait.

In this paper, a sequence of reanalyses and an operational analysis with horizontal resolutions ranging from ~ 60 to ~ 9 km, that all share a common model framework and data assimilation system, are used to characterize the variability in the wind field as observed by this weather station. The approach used in this paper represents an improvement over previous studies of the impact of model resolution on the representation of topographic flow distortion that have used reanalyses with different data assimilation systems and model parameterizations^22–24^. Please refer to the Methods Section for more information of the AWS data and the model suite used in this paper.

## Results

**Figure 3:**
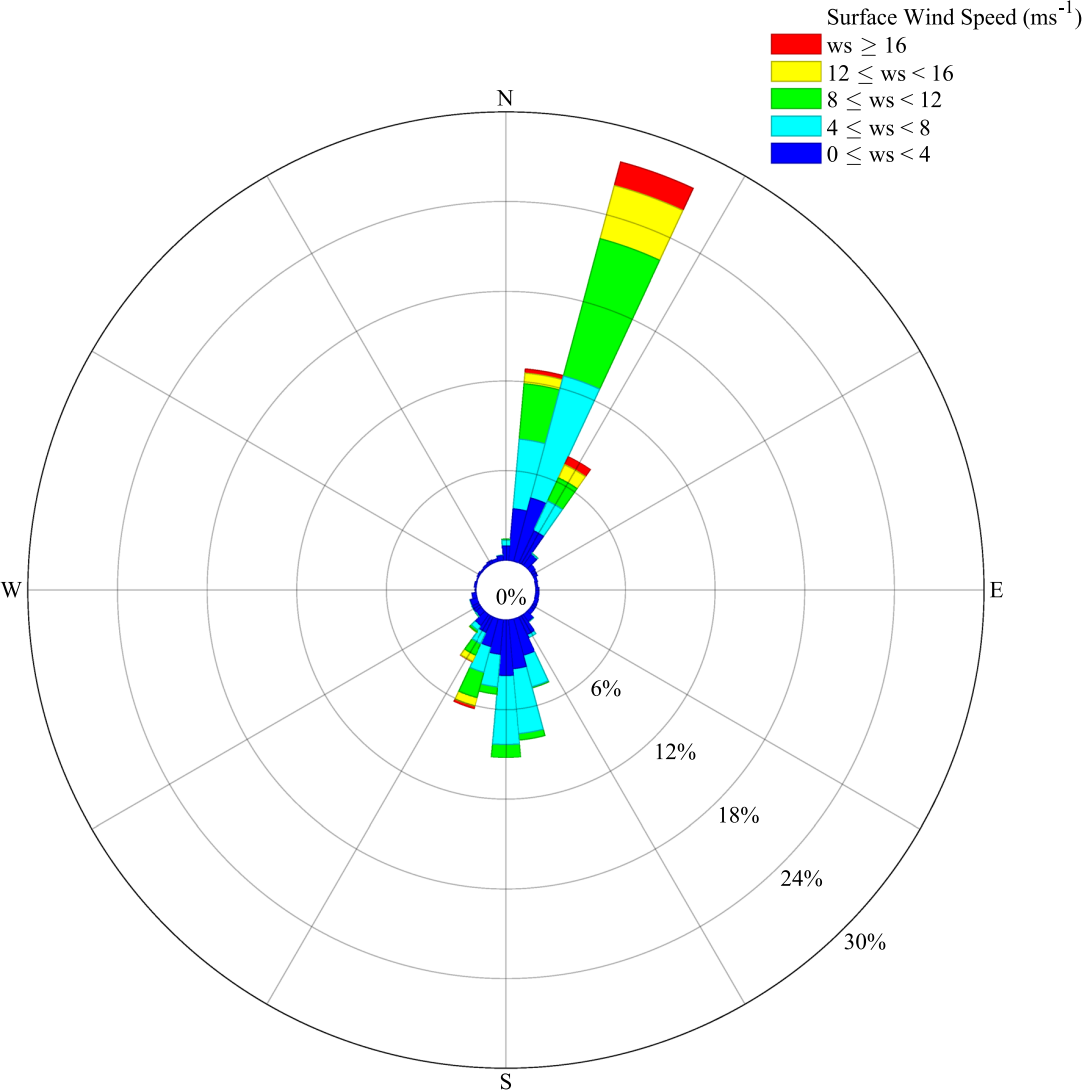
The wind rose from the Hans Island AWS Sept 2 2014 to Sept 30 2020 excluding May–July 2016.

Figure 3 presents the wind rose for the Hans Island AWS data. The tendency for bidirectional flow, in the NNE or SSW directions, is apparent as is the preference for northerly flow. This directionality is consistent with the orientation of the Kennedy Channel in the vicinity of Hans Island (Fig. 2). The mean wind speed is  5.8 ms^−1^ with a 99th percentile wind speed of 18.7 ms^−1^. For northerly flow, the maximum wind speed was 29.4 ms^−1^ with a 99th percentile wind speed of 19.7 ms^−1^. The corresponding values for southerly flow were 22.4 ms^−1^ and 13.9 ms^−1^ respectively.

**Figure 4:**
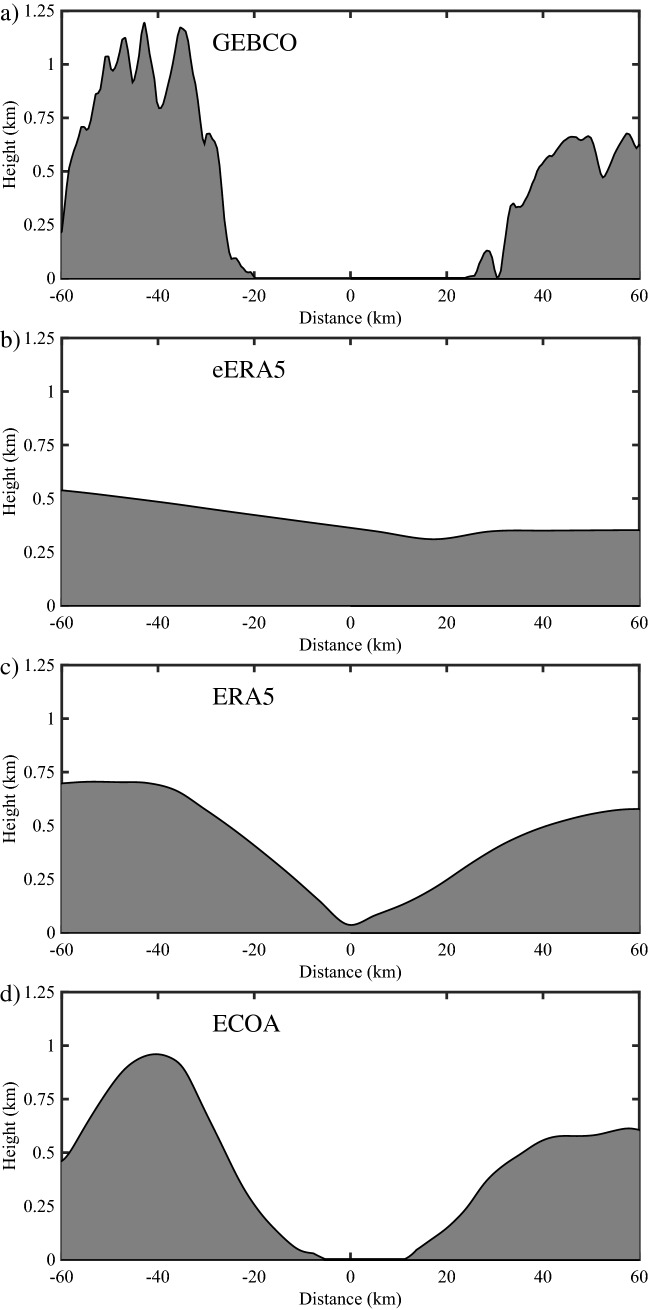
Cross-section through the topography normal to Nares Strat in the vicinity of Hans Island for the: **(a)** GEBCO digital elevation model, **(b)** eERA5; **(c)** ERA5 and **(d)** ECOA. Negative distances are to the northwest of Hans Island with positive distances to the southeast.

The narrowness and steep topography of Nares Strait plays an important role in the region’s wind climate. Before comparing the wind observations from Hans Island with those from the models, we show in Fig. 4, cross-sections of the topography normal to Nares Strait in the vicinity of Hans Island as represented in the: GEBCO 15 arc-second, here subsampled to 1 km, digital elevation model^25^; the eERA5 with a resolution of ~ 60 km; the ERA5 with a horizontal resolution of ~ 30 km and the ECOA with a horizontal resolution of ~ 9 km. The GEBCO cross-section (Fig, 4a) clearly represents the narrowness of Nares Strait in the vicinity of Hans Island as well as the steep topography along both sides of the strait. The eERA5 cross-section (Fig. 4b) has a very modest representation of Nares Strait as a broad minimum in topography. The ERA5 cross-section (Fig. 4c) is able to resolve more details of the strait but, for example, there is no open water present and the height of the topography along the sides of the strait is underestimated. The ECOA cross-section (Fig. 4d) approaches the level of detail in the GEBCO cross-section but is still unable to fully resolve the steepness or the narrowness of the strait.

**Figure 5:**
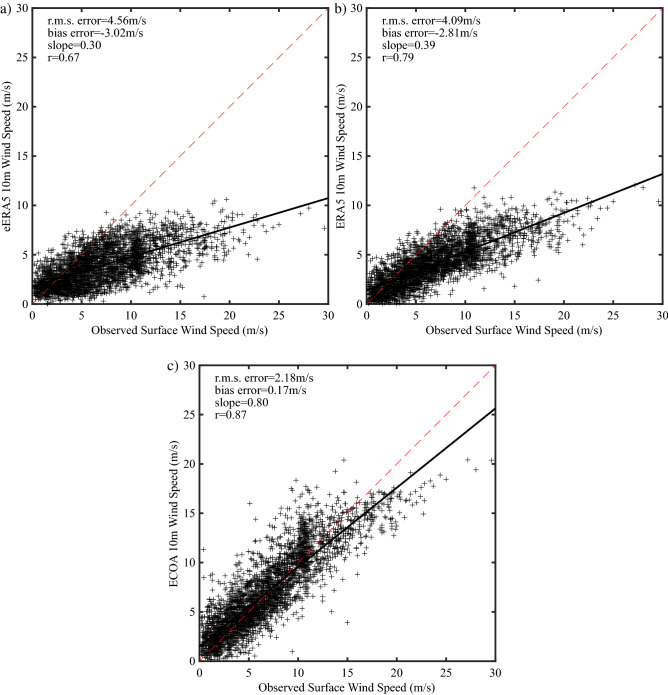
Scatterplots of the observed 6-hourly surface wind speed and the 10 m model wind speed at Hans Island. Results are shown for the: **(a)** eERA5, **(b)** ERA5 and **(c)** ECOA. Data from January 1 2016-September 30 2020 excluding May–July 2016 was used.

Figure 5 provides scatterplots of the observed surface wind speed at the AWS with the 10 m wind speeds from the three model datasets. Statistics that provide quantitative measures of the goodness of the fit are shown. These include the root-mean-square (rms) and bias errors as well as the least-squares slope and the correlation coefficient *r*. A least-squares slope of 1 implies a one-to-one relationship exists between the observations and model output.

The results show that both the eERA5 (Fig. 5a) and the ERA5 (Fig. 5b) have a marked tendency to underestimate the wind speed at the AWS site with the ERA5 having a slightly better fit. For example, the rms error for eERA5 is 4.56 ms^−1^ with a least-squares slope of 0.44; while for ERA5 the corresponding values are 4.09 ms^-1^ and 0.55. The bias error for both of these datasets was ~ −3 ms^−1^. By comparison, the ECOA (Fig. 5c) has an improved fit to the data with a rms error of 2.18 ms^−1^ and a bias error of 0.17 ms^−1^ as well as a reduced tendency to underestimate the wind speed at the AWS site indicated by the least-squares slope of 0.8. The correlation coefficient increases from 0.67 for the eERA5 to 0.79 for the ERA5 to 0.87 for the ECOA.

**Figure 6:**
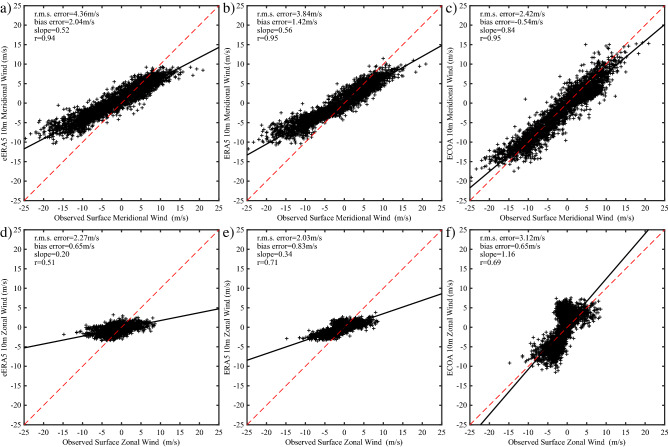
Scatterplots of the observed 6-hourly components of the surface wind and the components of the 10 m model wind at Hans Island. Results are shown for the meridional component for the: **(a)** eERA5, **(b)** ERA5 and **(c)** ECOA. Results are also shown for the zonal component for the: **(d)** eERA5, **(e)** ERA5 and **(f)** ECOA. Data from January 1 2016-September 30 2020 excluding May–July 2016 was used.

As noted above, the flow in the vicinity of Hans Island has a pronounced bidirectionality. Figure 6 shows the scatterplots of the observed meridional and zonal components at the AWS with the corresponding components of the 10 m wind from the three model datasets. In general, the characteristics noted above with respect to the 10 m wind speed hold for the components as well. In particular, there is a reduction in the rms and bias errors as well as an increase in the least-squares slopes as one transitions from the lower resolution to higher resolution datasets. In addition, the fit to the zonal component is generally poorer than that for the meridional component, a characteristic that is most noticeable for the eERA5 and ERA5 datasets.

**Figure 7:**
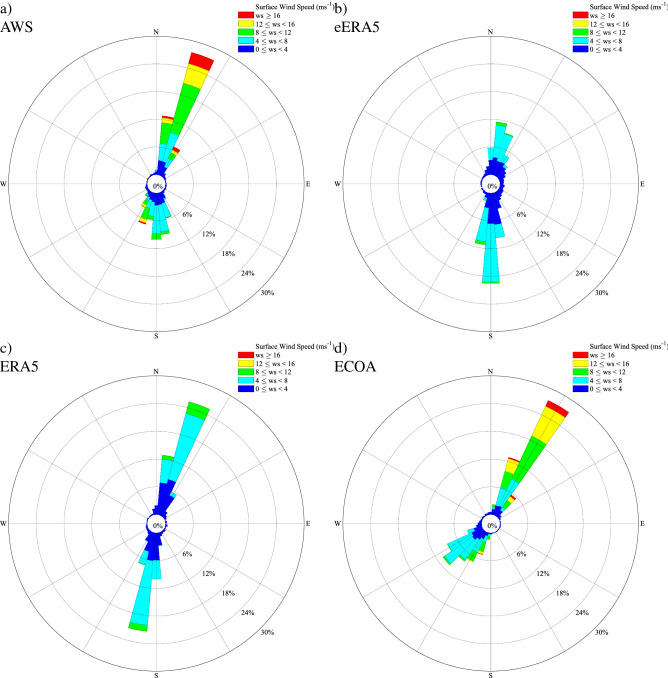
The wind rose at Hans Island for the: **(a)** AWS, **(b)** eERA5, **(c)** ERA5 and **(d)** ECOA Data from January 1 2016–September 30 2020 excluding May–July 2016 was used.

In Fig. 7 the wind rose at Hans Island from the three model data sets are compared to observations for the period of overlap. The characteristics are consistent with the results from the scatterplots. In particular, there is an increase in frequency of occurrence of high wind speeds as one transitions from the eERA5 to the ECOA. The above noted quantitively poorer fit in the zonal component in the eERA5 (Fig. 7b) and ERA5 (Fig. 7c) results in an inability of these models to capture the preference for NNE to SSW bidirectional flow. As compared to the observed wind rose (Fig. 7a), the ECOA wind rose (Fig. 7d) has a tendency to underestimate the magnitude of extreme wind speeds. There was also a slight discrepancy in the orientation of the winds in the ECOA as compared to observations.

**Figure 8:**
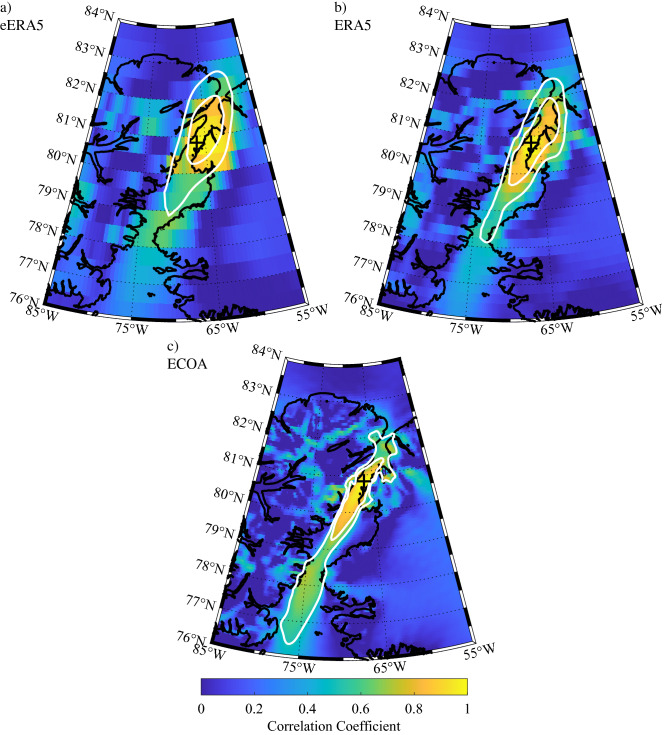
One-point correlation maps showing the correlation between the 10 m wind speed at Hans Island and at other gridpoints in the Nares Strait region as represented in the: **(a)** eERA5, **(b)** ERA5 and **(c)** ECOA for the period 2016–2020. The location of Hans Island is indicated by the ‘ + ’. The 0.5 and 0.7 correlation coefficient contours are shown in white. Figure created using Matlab R2019b (https://www.mathworks.com).

It is of interest to characterize the representativeness of the model wind field at Hans Island for flow along the entirety of Nares Strait. To accomplish this, one-point correlation maps^26^ of the 10 m wind speed at Hans Island with the wind speed at other grid points in the Nares Strait region were calculated for each of the model datasets (Fig. 8). Also shown is the 0.5 and 0.7 correlation coefficient contours that encompasses the area in which the variability in the 10 m wind speed at Hans Island can explain ~ 25% and ~ 50% of the variability in the 10 m wind speeds^27^. The 0.7 correlation coefficient contours were also fit to ellipses using a least-squares approach^28^ so as to allow for a determination of their eccentricity and orientation^29^.

The results for all three model data sets indicate a high degree of correlation in the wind speed along Nares Strait with that at Hans Island. For eERA5, the region with correlation coefficients greater then 0.5 and 0.7 extend over both Ellesmere Island and Greenland resulting, for example, in an elliptical fit to the r = 0.7 contour that has an eccentricity of 0.79 (Fig. 8a). This characteristic of the spatial correlation of the wind field, i.e. the correlation over the regions of high topography adjoining Nares Strait, is reduced in ERA5 resulting in an eccentricity of 0.95 for the r = 0.7 contour (Fig. 8b). In contrast, the 0.7 contour for the ECOA is, for the most part, restricted to Nares Strait resulting in an eccentricity of 0.98 (Fig. 8c). For all three datasets, there is an asymmetry in the extent of the region of elevated correlation with the Hans Island 10 m wind speed. In particular, the region of elevated correlation extends farther to the south as compared to the north. Indeed for the ECOA, the 10 m wind speeds as far south as Smith Sounds are correlated with that at Hans Island, at the r = 0.5 level.

**Figure 9:**
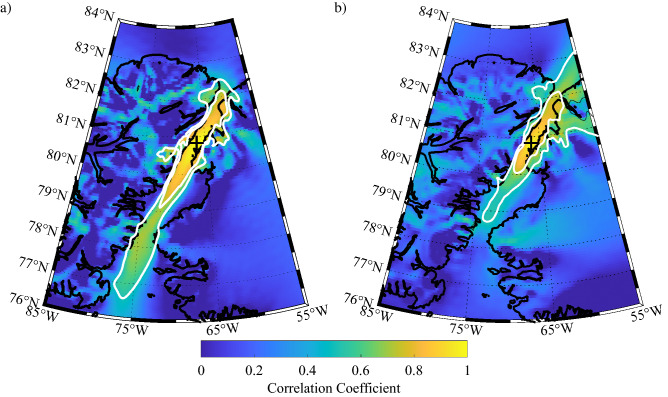
One-point correlation maps showing the correlation between the 10 m wind speed at Hans Island and at other gridpoints in the Nares Strait region as represented in the ECOA. Results are shown for times during the period 2016–2020 during which there was: **(a)** northerly and **(b)** southerly flow at Hans Island. The location of Hans Island is indicated by the ‘ + ’. The 0.5 and 0.7 correlation coefficient contours are shown in white. Figure created using Matlab R2019b (https://www.mathworks.com).

To test the hypothesis that this asymmetry is the result of the preference for northerly flow at Hans Island, the one-point correlation maps were recalculated for times in which either northerly or southerly flow were observed at Hans Island. Results are shown in Fig. 9 for the ECOA dataset. The one-point correlation map for northerly flow (Fig. 9a) is very similar to that when there is no preference to the wind direction (Fig. 8c). In contrast, the one-point correlation map for southerly flow (Fig. 9b) indicates that the region of elevated correlation extends over the southern Lincoln Sea as well as adjacent regions of north Greenland.

**Figure 10:**
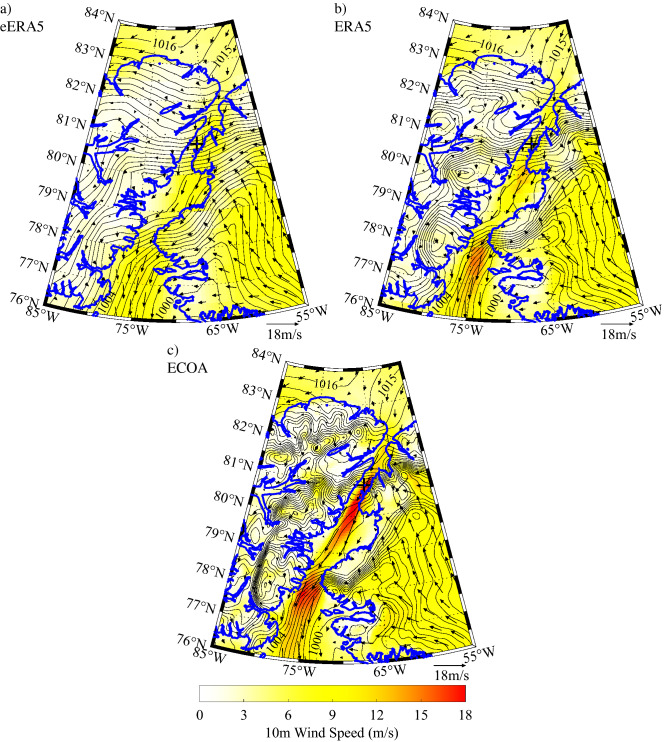
Composite sea-level pressure (contours-mb), 10 m wind speed (shading-ms^−1^) and 10 m wind (vectors-ms^−1^) for high speed northerly flow at Hans Island as represented in the: **(a)** eERA5, **(b)** ERA5 and **(c)** ECOA. Figure created using Matlab R2019b (https://www.mathworks.com).

**Figure 11:**
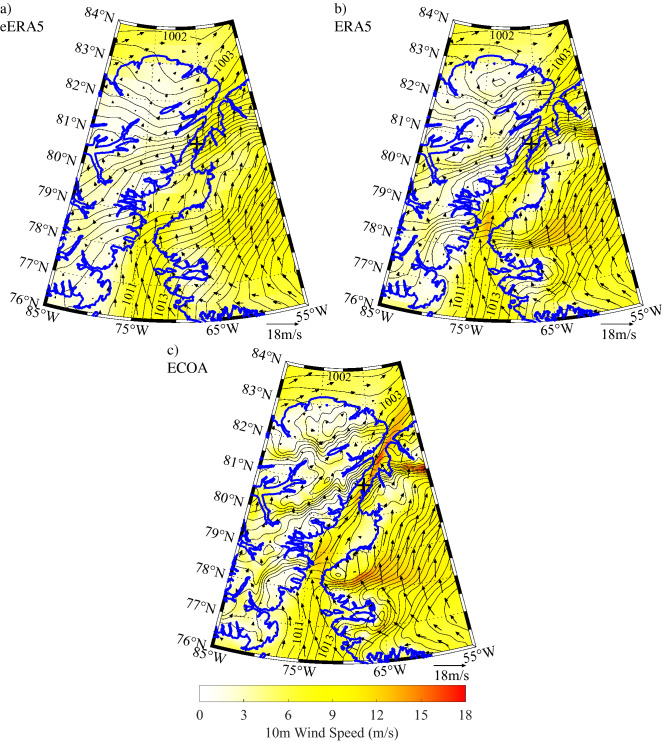
Composite sea-level pressure (contours-mb), 10 m wind speed (shading-ms^−1^) and 10 m wind (vectors-ms^−1^) for high speed southerly flow at Hans Island as represented in the: **(a)** eERA5, **(b)** ERA5 and **(c)** ECOA. Figure created using Matlab R2019b (https://www.mathworks.com).

We conclude the presentation of results with composites of the environmental conditions associated with extreme northerly and southerly flow events at Hans Island. This was accomplished by identifying the times when the observed wind speed at Hans Island exceeded the 99th percentile thresholds for northerly, 19.7 ms^−1^, and southerly, 13.9 ms^−1^ winds. For each event, only the time at which the wind speed was a maximum was included. As a result, there were 16 distinct northerly events and 15 distinct southerly events. For these times, the sea-level pressure, 10 m wind speed and 10 m wind fields from the 3 model datasets were averaged resulting in composites shown in Figs. 10 and 11.

The composite extreme northerly wind event at Hans Island (Fig. 10) is associated with higher sea-level pressures over the Lincoln Sea and lower sea-level pressures over northern Baffin Bay. The higher wind speeds are focused in the region to the south of Hans Island and generally increase from the eERA5 (Fig. 10a) to the ERA5 (Fig. 10b) to the ECOA (10c). In a similar vein, there is an increase in the localized gradients in sea-level pressure along the strait as well as over northern Baffin Bay as model resolution increases.

In contrast, the composite extreme southerly wind event at Hans Island (Fig. 11) is associated with a reversal in the meridional pressure gradient between the Lincoln Sea and northern Baffin Bay. In this case, the highest wind speeds tended to be to the north of Hans Island with a similar increase in wind speed and higher gradients in sea-level pressure with resolution. There was however also evidence of elevated winds over the southern Kane Basin in both the ERA5 (Fig. 11b) and the ECOA (Fig. 11c) that were absent in the eERA5 (Fig. 11a).

## Discussion

The Nares Strait wind field plays an important role in modulating the export of thick multi-year sea ice from the Last Ice Area situated to its north as well as in maintaining the NOW located at its southern end^1,9^. There has been a significant loss of multi-year ice from the Arctic in recent years^14^ as well as an increase in ice export along Nares Strait^7^ and there is also evidence that the NOW is increasing in area^10^. The remoteness of the Nares Strait region has limited our ability to characterize the wind field thereby leading to uncertainty in its role in the changes to the ice that are now being observed in the region. The steep and narrow nature of the strait (Fig. 1) leads to high winds arising from interactions with the topography along its coast that remain a challenge for models to capture^1,18^. In this paper, we have used wind observations from an automatic weather station situated on Hans Island, in the middle of Nares Strait (Figs. 1, 2), to document the variability in wind field along the strait and the ability of numerical models to represent this variability.

The Hans Island wind observations confirm that the flow along the strait is bidirectional, with preferred directions from the NNE or SSW and that northerly flow is most common (Fig. 3). There is evidence that extreme events with either directionality occur with wind speeds in excess of 20 ms^−1^. One such northerly wind event destroyed an ice camp that had been established along the Kennedy Channel in April 2005^17^.

To assess the role that horizontal resolution plays in the ability of models to represent the variability in the wind observed at Hans Island, three different versions of the ECMWF’s IFS were considered with resolutions of ~ 60 km (eERA5), ~ 30 km (ERA5) and ~ 9 km (ECOA). The common lineage of the models allows one to control for biases arising from difference in model architecture and parameterizations^22–24^.

The comparison of cross-sections through the reference and model topographies (Fig. 4) clearly shows the improvement in representation of the narrowness and steepness of the strait in the vicinity of Hand Island in the ECOA as compared to the eERA5 and ERA5.

The comparison for the 10 m wind speed (Fig. 5) shows a measurable improvement in the representation of the observations with increasing model resolution with the largest jump occurring between the ERA5 (resolution ~ 30 km) and the ECOA (resolution ~ 9 km). For example, the rms error decreased from 4.09 ms^−1^ for the ERA5 to 2.18 ms^−1^ for the ECOA; while the slope of the least squares linear fit increased from 0.39 for the ERA5 to 0.8 for the ECOA. The latter characteristic implies that the eERA5 and the ERA5 have a tendency to underestimate the wind speed during high wind speed events to a greater extent than the ECOA.

This may be related to the inability of these models to represent the open water in Nares Strait and its replacement with land that generally has higher surface roughness (Fig. 4). Nevertheless, all three versions of the IFS have relatively high correlation coefficients, varying from 0.67 for the eERA5 to 0.87 for the ECOA, suggesting that a degree of linearity exists between model and observations.

The comparison with the components of the wind (Fig. 6) shows the same improvement with increasing resolution with the fit to the meridional component being systematically better than the zonal component for the eERA5 and ERA5. To first order, the winds along Nares Strait are the result of the difference in sea-level pressure between the Lincoln Sea and northern Baffin Bay^1^. All the models, to a greater or lesser extent, represent the narrowness of the strait and hence the tendency for the flow to be ageostrophic and controlled by the pressure gradient in the along-strait, i.e. meridional, direction. The composite high speed events (Figs. 10 and 11) clearly show that the magnitude of these along-strait gradients are a function of resolution. In contrast, the zonal component of the wind along Nares Strait is constrained by a model’s ability to represent the gradient in topography, that is largest in the zonal direction (Figs. 1 and 4). The poorer fit of this component of the wind for the eERA5 and ERA5 is associated with the inability of these models to represent this gradient.

The comparisons of model wind roses with observations (Fig. 7) are consistent with the above discussion and in particular highlight the tendency for the lower resolution eERA5 and ERA5 datasets (Fig. 7b, b) to have the winds aligned in the meridional direction as compared to the NNE to SSW direction in the ECOA dataset (Fig. 7d) and in the AWS data (Fig. 7a).

The one-point correlation maps of the 10 m wind field (Fig. 8) confirm this characteristic in that both the eERA5 and ERA5 have regions of high correlation that spill out over the topography along the strait. In contrast for the ECOA, the region of high correlation is restricted, for the most part, to Nares Strait. The eccentricities for the elliptical fits to the r = 0.7 contour, that range from 0.79 for the eERA5 to 0.98 for the ECOA provide a quantitative confirmation of this characteristic. The one-point correlation maps for the ECOA 10 m wind (Figs. 8c and 9) confirm that the Hans Island observations are representative of variability in the wind field along Nares Strait and, for southerly flow, over the southern Lincoln Sea as well.

The structure of the composite extreme wind speed events (Figs. 10 and 11) are consistent with the other results of this study and confirm the requirement of high horizontal resolution to resolve the impact that the topography of the Nares Strait region has on the wind field. In particular, high model resolution is required to resolve the localized large spatial gradients in the along strait sea-level pressure field that is responsible for the ageostrophic nature of the flow. Smith Sound as well as the Kennedy and Robeson Channels are regions where these large gradients occur. Both Smith Sound and the Kennedy Channel are regions where high speed northerly and southerly wind events occur. This confirms historical observations made during the nineteenth century^16,19,20^.

The results of this study suggest that a model resolution of at least 9 km is required to represent the wind field along Nares Strait providing a confirmation of earlier work^18^ in which a 6 km limited area numerical weather prediction model was used to develop a 2-year climatology of the regional wind field as well as the study^30^ that used the 9 km ECOA data to represent air-sea interaction over the NOW. It also suggests that climatologies^9,31,32^ and ice/ocean models^33–35^ of Nares Strait that are based on or forced by atmospheric datasets with horizontal resolutions coarser than 9 km may underestimate wind-driven processes active in the region. In particular, the coarser resolution datasets may not represent the narrowness and steepness of the strait leading to an underestimation of the along-strait pressure gradients responsible for the ageostrophic nature of the flow^2^. In addition, the coarser resolution datasets may not be able to resolve the open water of the strait replacing it with land (Fig. 4) that has a higher surface roughness leading to an underestimation in the surface wind speeds. This study also stresses the importance of the Hans Island site, situated within Nares Strait, to validate model performance along the strait.

## Methods

The Hans Island AWS was installed by a joint American, Canadian, Danish and U.K. team in May 2008^21^. The surface air temperature, wind speed and direction as well as solar radiation and surface pressure are available starting in September 2014 at a 30 min interval. There are numerous data gaps as well as a period of approximately 3 months, May–July, during 2016 when the sign of the zonal wind component was intermittently reversed. For this study, we will use the wind speed and direction data for the period September 2 2014-September 30 2020 with the exception of the 3 month period with bad wind direction data during 2016. The data was subsampled to a 6-hourly interval providing data at 00, 06, 12 and 18 GMT.

In this paper, we will make use of 3 model datasets based on the ECMWF’s Integrated Forecast System (IFS). Included is the new fifth generation reanalysis from the ECMWF or ERA5 with a horizontal resolution of ~ 30 km, as well as its ensemble version, eERA5 with a horizontal resolution of ~ 60km^36^ and the current version of their operational analysis or ECOA, with a horizontal resolution of ~ 9km^37^. The eERA5 ensemble consists of an unperturbed member and 9 perturbed members. For this work, we made use of the unperturbed member of the ensemble. For this study, we used the model data for the period 2016–2020. The eERA5 and ERA5 datasets are based on Cycle 41r2 of the IFS. Being an operational product, the ECOA data is based on a number of different cycles of the IFS from Cycle 41r2 up to Cycle47r1. No material changes to the IFS, that would impact the present study, occurred over the period 2016–2020. All three datasets use the same 4Dvar data assimilation system^38^, as well as same number of levels in the vertical, 137 up to 1 mb^36,39^.

For this study, the instantaneous 6-hourly 10 m winds and 100 m from these three datasets were interpolated to the location of Hans Island and the data was used at those times for which there are observations. A number of different interpolation schemes were used and the results were independent of scheme. For simplicity, the results shown are for a bilinear interpolation scheme. In total, ~ 550 days worth of data was included in this study. The AWS was situated at a height of approximately 170 m above sea level^21^.

## Data Availability

The author would like to thank the Scottish Association for Marine Sciences for access to the Hans Island automatic weather station data (https://dataservices.sams.ac.uk/aws/), Copernicus Data Services for access to the eERA5 and ERA5 data (https://cds.climate.copernicus.eu) and the National Center for Atmospheric Research for access to the ECOA data (https://rda.ucar.edu).
